# P-2160. Where Does Karius Testing Have a Clinical Impact? A Retrospective Analysis of Next Generation Sequencing

**DOI:** 10.1093/ofid/ofae631.2314

**Published:** 2025-01-29

**Authors:** Antonio Prado, Elise Ewens, Emily Shephard, Hita Bhagat, Regina Won

**Affiliations:** Legacy Emanuel Medical Center, Portland, Oregon; Legacy Emanuel Medical Center, Portland, Oregon; Legacy Health, Portland, Oregon; Legacy Health, Portland, Oregon; Legacy Medical Group, Gresham, Oregon

## Abstract

**Background:**

Karius testing, a commercially available microbial cell-free DNA assay, has emerged as a new diagnostic tool for clinicians, particularly for management of culture negative infections and in immunocompromised patients. There remains uncertainty regarding the clinical impact of next-generation sequencing in patient management.

**Figure:**
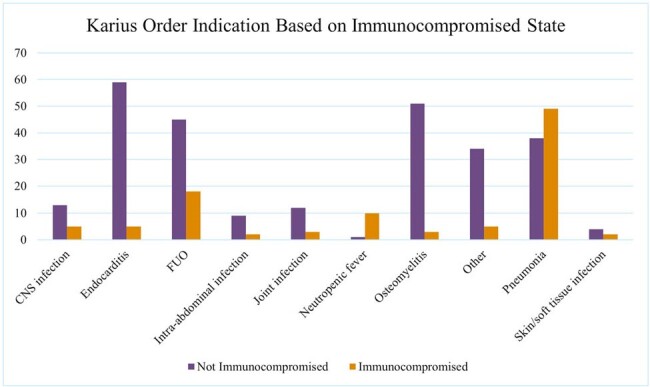

Figure 1

**Methods:**

This was a retrospective multi-site observational cohort study over a five-year period from 2018 to 2023 at a community hospital system. Patients were included if they had a Karius test ordered with results available to treating clinicians during the time period. The primary outcome was a change in clinical management defined as either the addition or removal of antibiotics, based on results from Karius testing.

**Figure:**
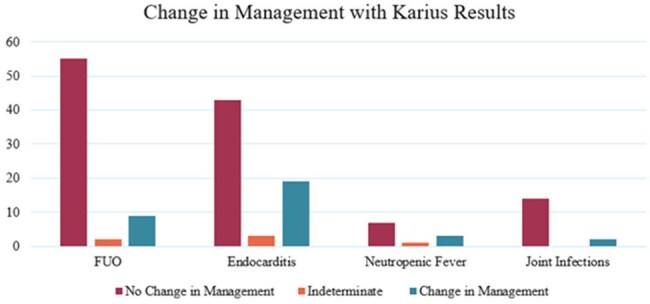

**Results:**

391 patients met inclusion criteria, with 283 (72%) in immunocompetent and 108 (28%) in immunocompromised patients. An infectious diseases (ID) consult was obtained in 355 (91%) cases. Fifty-two percent of Karius tests were positive, with an average of 1.1 pathogens reported. Concordance rate between positive cultures and Karius results was 12%. Pneumonia (24%), fever of unknown origin (FUO) (18%), and endocarditis (17%) were the most common indications for Karius orders (Fig 1). Testing changed management in 19% of all included individuals, and in 18% of immunocompromised patients. Karius testing was most impactful in neutropenic fever and endocarditis with 27% and 22% respectively changing management and least impactful when ordered for joint infection, where 12% resulted in a change in management (Fig 2).

**Conclusion:**

The Karius test changed antibiotic treatment in 19% of patients. It was most clinically relevant in neutropenic fever and endocarditis management. Even with our sizeable sample population including both immunocompromised and immunocompetent patients across a large, multi-site community hospital system, findings remained consistent compared to other smaller studies. Further investigation would be beneficial to determine how best to incorporate this emerging tool in clinical practice.

**Disclosures:**

All Authors: No reported disclosures

